# Supplementary material for: Fibroblast Activation Protein Targeted Photodynamic Therapy Selectively Kills Activated Skin Fibroblasts from Systemic Sclerosis Patients and Prevents Tissue Contraction
Source: Int J Mol Sci. 2021 Nov 24;22(23):12681. doi: 10.3390/ijms222312681 (PMC8657852; doi:10.3390/ijms222312681)
Supplement: Supplementary file 1 [file ijms-22-12681-s001.zip › ijms-1424065-supplementary.pdf]

Supplemental Figure S1: Analysis of *FAP* expression in lesional versus unaffected SSc skin.

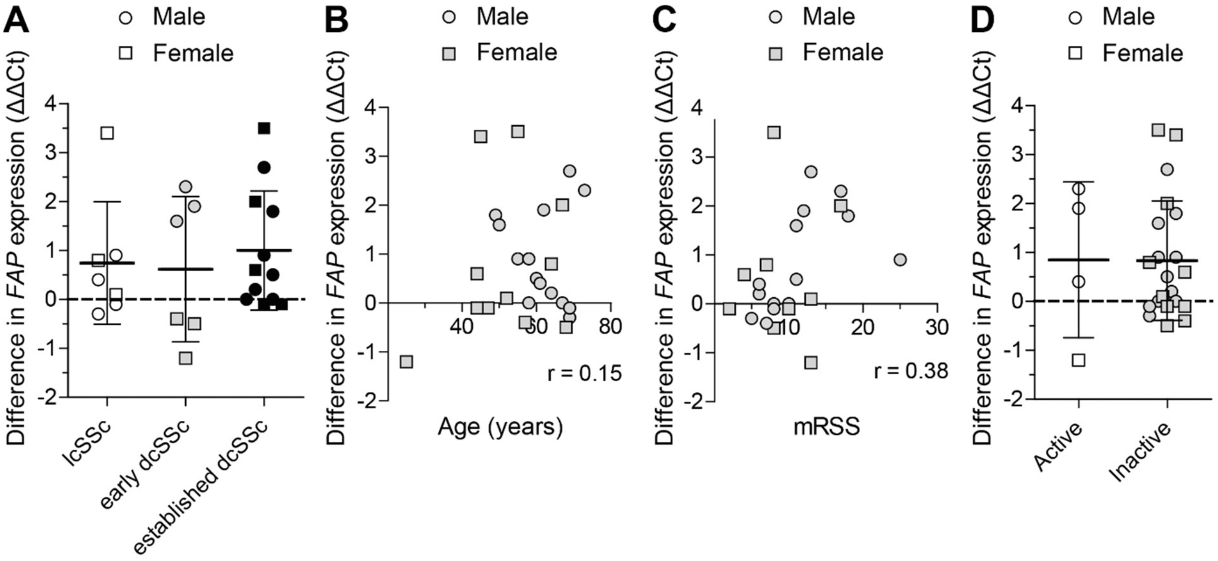

In this figure the difference in *FAP* expression between lesional and unaffected skin is plotted as ΔΔCt. A positive value means more *FAP* expression in lesional skin than in unaffected skin. In all panels males (14/25) were plotted as circles, and females (11/25) as squares. Panel **A** shows the difference in *FAP* expression in various SSc subtypes; limited cutaneous SSc (LcSSc), early diffuse cutaneous SSc (less than 2 years after diagnosis) and established diffuse cutaneous SSc (more than 2 years after diagnosis). There is no significant difference between these subtypes and difference in *FAP* expression. Panel **B** shows the correlation between patient age and difference in *FAP* expression. The Pearson's r was 0.15 and no significant correlation was observed. Panel **C** shows the correlation between a patient's modified Rodnan skin score and their difference in *FAP* expression. The Pearson's r was 0.38 and no significant correlation was observed. Of note, there were 2 missing values. Panel **D** shows difference in *FAP* expression in active versus inactive disease. Active disease was defined as an increase in mRSS in the past 6 months. No significant difference was observed between both groups.
